# Ankylosing Spondylitis Patients Commencing Biologic Therapy Have High Baseline Levels of Comorbidity: A Report from the Australian Rheumatology Association Database

**DOI:** 10.1155/2009/268569

**Published:** 2009-08-02

**Authors:** John Oldroyd, Lionel Schachna, Rachelle Buchbinder, Margaret Staples, Bridie Murphy, Molly Bond, Andrew Briggs, Marissa Lassere, Lyn March

**Affiliations:** ^1^Monash Department of Clinical Epidemiology, Cabrini Institute, 183 Wattletree Road, Malvern, VIC 3144, Australia; ^2^Department of Epidemiology and Preventive Medicine, School of Public Health and Preventive Medicine, Monash University, VIC 3004, Australia; ^3^Austin Spondylitis Clinic, Austin Health, Studley Road, Heidelberg, VIC 3084, Australia; ^4^Centre of Clinical Research Excellence in Therapeutics, Department of Epidemiology and Preventive Medicine, School of Public Health and Preventive Medicine, Monash University, VIC 3800, Australia; ^5^School of Physiotherapy, Curtin University of Technology, WA 6000, Australia; ^6^Department of Rheumatology, St George Hospital, NSW 2217, Australia; ^7^Department of Rheumatology, Royal North Shore Hospital, NSW 2065, Australia; ^8^Institute of Bone and Joint Research, University of Sydney, NSW 2065, Australia

## Abstract

*Aims*. To compare the baseline characteristics of a population-based cohort of patients with ankylosing spondylitis (AS) commencing biological therapy to the reported characteristics of bDMARD randomised controlled trials (RCTs) participants. *Methods*. Descriptive analysis of AS participants in the Australian Rheumatology Association Database (ARAD) who were commencing bDMARD therapy. *Results*. Up to December 2008, 389 patients with AS were enrolled in ARAD. 354 (91.0%) had taken bDMARDs at some time, and 198 (55.9%) completed their entry questionnaire prior to or within 6 months of commencing bDMARDs. 131 (66.1%) had at least one comorbid condition, and 24 (6.8%) had a previous malignancy (15 nonmelanoma skin, 4 melanoma, 2 prostate, 1 breast, cervix, and bowel). Compared with RCT participants, ARAD participants were older, had longer disease duration and higher baseline disease activity. *Conclusions*. AS patients commencing bDMARDs in routine care are significantly different to RCT participants and have significant baseline comorbidities.

## 1. Introduction

Ankylosing spondylitis is a chronic inflammatory rheumatic disorder predominantly affecting the spine with a reported prevalence in Caucasian populations varying between 0.05% to 0.23% [1]. AS has a substantial impact upon physical and emotional functioning and is associated with a progressive decline in quality of life [2, 3]. In those with severe disease, mortality is increased [4]. Extraspinal features may include peripheral arthritis, uveitis, enteritis, and psoriasis. Traditional disease modifying antirheumatic drugs (DMARDs) have been ineffective for the spinal component of the disease with the mainstay of treatment consisting of exercise therapy [5] and nonsteroidal anti-inflammatory drugs [6, 7].

Recently, biological disease modifying antirheumatic drugs (bDMARDs) including the tumor necrosis factor (TNF alpha) inhibitors etanercept, infliximab, and adalimumab have been shown to reduce disease activity and results in improvements in pain, function, and quality of life in AS [8–12]. Randomized controlled trials (RCTs) have established large treatment benefits for spinal pain, mobility, and function as well as improvements in extraspinal features [9, 10, 13]. Whether these effects will be fully realized and maintained in routine care remains to be determined as the trials have been of relatively short duration (e.g., 24–54 weeks) and have excluded important patient groups (i.e., patients under the age of 18 years and those with a history of cardiac, renal, hepatic, psychiatric, or neurologic disease or history of malignancy); and baseline comorbidities of trial participants have not been reported in detail [11, 14–16].

To address the limitations of clinical trials and determine the long-term safety and effectiveness of bDMARD therapy in AS in a “real life” context, population-based registries that allow careful longitudinal observation in routine clinical care have been established in several countries. These include the Australian Rheumatology Association Database (ARAD) [17], the British Society for Rheumatology Biologics Registry (BSRBR) [18], the Spanish Registry for adverse events of biological therapies in Rheumatic Diseases (BIOBADASER) [19], the Danish Database for Biological Therapies (DANBIO) [20], the National Register for Biological Treatment in Finland (ROB-FIN) [21], and the Norway Disease Modifying AntiRheumatic Drugs Register (NOR-DMARD) [22].

In determining the long-term safety and effectiveness of bDMARDs for AS, it is important to consider the potential confounding effects of other comorbid conditions. Unless comorbid conditions are considered, adverse events may be incorrectly attributed to bDMARD therapy. Comorbidity has been shown to be a significant predictor of health outcomes in patients with rheumatoid arthritis [23, 24], with premature mortality largely attributed to cardiovascular disease, infection, and malignancy [25]. Several registries, including our own, have reported a high prevalence of comorbid conditions in patients with rheumatoid arthritis commencing bDMARDs [20, 26, 27] but the prevalence of comorbid conditions in patients with AS commencing bDMARD therapy has not, thus far, been widely reported.

Before bDMARDs can be prescribed under government-subsidized schemes in Australia certain stringent criteria must be met. These include fulfilment of the modified New York criteria for definite AS [28] and failure to respond, over a three-month period, to two or more NSAIDs and an exercise program. Failure to respond is defined as a Bath Ankylosing Spondylitis Disease Activity Index (BASDAI) of at least 4.0 and raised inflammatory markers (erythrocyte sedimentation rate (ESR) >25 mm/hour and/or C-reactive protein (CRP) >10.0 mg/L) [29]. Similarly restrictive criteria exist in the UK [30].

In contrast to some other countries [31], previous and/or current malignancy is not an absolute contraindication to prescribing bDMARDs in Australia although increased vigilance is recommended for patients who have a history of malignancy [32]. The incidence and types of malignancies that occur in Australia differ somewhat from those reported elsewhere providing an added imperative to collect Australian data rather than rely on the observations from other registries. For example, Australia has the highest incidence of both melanoma and nonmelanoma skin cancers (NMSCs) in the world attributed to the outdoor lifestyle of a predominantly fair-skinned population and more recently to the reduced ozone layer in this region [33, 34]. It also has a comparatively higher risk of skin cancer in organ transplant recipients who also receive immunosuppressive agents [35] and we have previously reported a threefold increase in the risk of melanoma in Australian methotrexate-treated patients with rheumatoid arthritis compared with the general population [36].

The aim of this study was to describe the baseline characteristics including comorbidities of a population-based cohort of Australian patients with AS participating in ARAD who are commencing bDMARDs and to compare them to the baseline characteristics of participants enrolled in published bDMARD RCTs in AS.

## 2. Methods

### 2.1. ARAD Design

The structure and governance of ARAD has been described previously [17]. Briefly, ARAD was established by the Australian Rheumatology Association (ARA) in 2003 as a voluntary registry to collect longitudinal health outcomes data from Australian patients with inflammatory arthritis treated with bDMARDs. Recruitment of controls (those not prescribed biologics) commenced in 2000 and is ongoing although numbers are small to date. The primary goal of ARAD is to determine the long-term effectiveness and safety of bDMARDs in routine clinical practice.

Ethical approval has been granted by the Institutional Human Research Ethics Committees of Cabrini Hospital, Melbourne; Northern Sydney Health; South Eastern Sydney and Illawarra Area Health; Australian Government Department of Veterans Affairs; Monash University; Royal Children's Hospital, Melbourne; NSW Population and Health Services Research Ethics; Tasmanian Human Research Ethics Committee; Australian Institute of Health and Welfare; Cancer Institute of NSW; Department of Health (CHIC) Western Australia; St Vincent's Hospital, Melbourne; The Children's Hospital, Westmead; Department of Health South Australia Human Research Ethics Committee; Queensland Cancer Registry; the Tasmanian Cancer Registry; the Victorian Cancer Registry; the ACT Cancer Registry; Northern Territory Cancer Registry, the New South Wales Central Cancer Registry, the Western Australian Cancer Registry, and the National Cancer Statistics Clearing House. All participants provide written informed consent.

### 2.2. Data Collection

Data collected from the rheumatologist at baseline includes the TNF alpha inhibitor prescribed, ESR, CRP, and the Bath Ankylosing Spondylitis Disease Activity Index (BASDAI) [37]. The BASDAI is a quick, reliable, self-administered instrument used to determine disease activity in patients with AS. It measures severity of fatigue, spinal and peripheral joint pain, localized tenderness, and morning stiffness and is scored from 0 to 10 (0 = best).

All ARAD participants complete a detailed entry questionnaire and six-month followup questionnaires returned in a reply paid envelope. Returned data are scanned into the database and subject to rigorous quality control and data validation processes to ensure database quality. Data collected from the participants include: demographic details, disease duration and severity, self-reported past and current medical history including cancers and other chronic conditions, use of antirheumatic drugs, smoking, and alcohol history, generic measures of quality of life including the Short Form-36 (SF-36)(subscale scores range 0–100, 100 = perfect health) [38], and Assessment of Quality of Life (AQoL) (score range 0-1, 1 = full health) [39], and arthritis-specific disability assessed by the Health Assessment Questionnaire modified for spondyloarthropathies (S-HAQ) (score range 0–3, 0 = no disability) [40].

For the purpose of this study, the baseline characteristics and health-related quality of life of AS patients commencing bDMARDS were extracted from the last questionnaire completed prior to commencing bDMARD therapy. For participants who enrolled in ARAD after starting therapy, BASDAI, ESR, and CRP at the time of starting therapy were obtained from the referring rheumatologist and comorbidities were only considered if data were available either prior to or within 6 months of the start of bDMARDS. Health-related quality of life data prior to commencing bDMARD therapy was unavailable for these participants.

### 2.3. Verification of Malignancy

We verified all self-reported malignancies identified prior to commencement of bDMARDs by record linkage to the National Cancer Statistics Clearing House (NCSCH) and the Victorian State Cancer Registry (VCR) in 2007. The NCSCH is a repository of data for each state cancer registry in Australia apart from the state of Victoria. Since 1982 the state registries have collected details of all malignancies apart from nonmelanocytic skin cancers, and notification of malignancy to the relevant state registry is mandatory by law. Virtual complete ascertainment is achieved by notification from pathology laboratories, hospital medical record departments, and by screening of death certificates. The International Classification of Diseases—9th Revision (ICD-9) is used to code site of malignancy [41] and the ICD-O morphology rubrics to code histological type [42, 43]. At the time of the study the NCSCH and VCR were complete up until the end of 2003 and 2005, respectively. Self-reported malignancies prior to 1982 and after 2003/2005 for the NCSCH and VCR, respectively, and all self-reported nonmelanocytic skin cancers were verified by obtaining pathology reports and/or confirmation by the treating doctor.

### 2.4. Identification of Published AS RCTs of bDMARDs

A PubMed search was conducted using the terms “ankylosing spondylitis” and the names of the three TNF inhibitors individually “infliximab” or “etanercept” or “adalimumab” to identify relevant AS RCTs of bDMARDs. Relevant baseline participant data were extracted from the RCTs and weighted means calculated for comparison with the ARAD data.

### 2.5. Data Analysis

Responses to the health related quality of life instruments (SF-36, EuroQoL and AQoL) were coded according to the standard published algorithms as described by the developers [38, 39, 44]. Independent sample *t*-tests were used to compare data in ARAD with that reported in previously conducted RCTs. All data were analysed using STATA (Version 10.0, Stata Corporation, College Station, Tex, USA).

## 3. Results

To 11th December 2008, 3025 participants were enrolled in ARAD. They included 2366 with rheumatoid arthritis, 389 with AS, 186 with psoriatic arthritis, and 83 with juvenile idiopathic arthritis. 201 (75.2%) rheumatologists from all Australian states and territories had contributed patients. Thirty-five out of the 389 AS participants (9%) had never taken bDMARD therapy and were excluded from further analysis. At the time of the analysis, 136 were currently taking etanercept, 124 were taking infliximab, 60 were taking adalimumab (*n* = 320), and 34 were not currently taking bDMARDs.

The baseline characteristics of the 354 AS participants who commenced biological therapy are summarised in Table 1. 

At the time of commencement of bDMARD therapy, 72 (20.3%) participants were taking at least one DMARD (65 were taking one DMARD and 7 were taking two), most commonly methotrexate (*n* = 50, 14.1%) or salazopyrin (*n* = 22, 6.2%); while 42 (11.9%) participants were taking prednisolone. Participants had evidence of active disease (mean (SD) BASDAI score 7.6 (4.5). Of those with AS who had ever taken bDMARDs (*n* = 354), quality of life data at baseline was available for 198 (56%) who had enrolled in ARAD prior to or within 6 months of commencing bDMARDs. They had moderate disability (mean (SD) S-HAQ 0.86 (0.60) and impaired quality of life, mean (SD) AQoL score 0.55 (0.25); SF-36 Physical Component score 36.2 (10.6), SF-36 Mental Component score 45.1 (11.1)).

**Figure 1:**
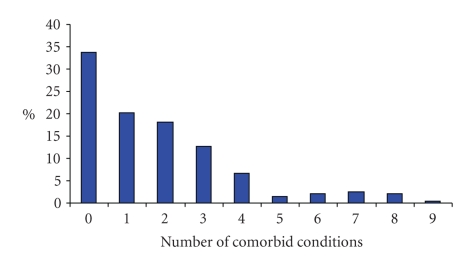
Number of comorbid conditions in patients with ankylosing spondylitis commencing biological therapy (*n* = 198).

At least one comorbid condition (past or current) was reported by 131 (66.1%) participants and 91 (46.0%) reported more than one (Figure 1).

The most frequently self-reported comorbidities were gastrointestinal disease reported by 61 (31.3%) participants, hypertension 51 (25.8%), eye disorders 32 (16.1%), dyslipidaemia 31 (15.6%), and depression 28 (14.1%) (Table 2).

Twenty four participants (6.8%) had a previous malignancy: nonmelanoma skin cancer (*n* = 15), melanoma (*n* = 4), prostate cancer (*n* = 2), breast cancer (*n* = 1), cervical cancer (*n* = 1), and bowel cancer (*n* = 1).

We identified 4 published RCTs of bDMARDs in AS (Table 3) [11, 14–16]. Compared with participants in previous RCTs, there was a similar proportion of males (71.8% versus 73.8%, *P* = .46), but ARAD participants were older (mean (SD) age 45.1 (12.3) years versus 41.9 (6.0) years, *P* < .001), had a longer disease duration (mean (SD) duration 18.5 (12.1) years versus 12.6 (5.0) years, *P* < .001) and had higher baseline BASDAI scores (mean (SD) 7.6 (4.5) versus 4.1 (0.83), *P* < .001).

## 4. Discussion

We have described the baseline comorbidities of a population-based cohort of patients with AS commencing biologic therapy extracted from a national biologic registry. As 75% of Australian rheumatologists contribute patients to ARAD and as all states and territories are represented, we think that our patient sample is representative of the Australian population. Two thirds of our sample reported having at least one comorbid condition, almost half (46.0%) had more than one, and 6.8% had a history of malignancy. In comparison with participants in RCTs of bDMARDs for AS, ARAD AS participants commencing biological therapy were older, had longer duration of disease, and had more severe disease at the time of commencement of bDMARDs. They also had significant disability and impaired quality of life.

**Figure 2:**
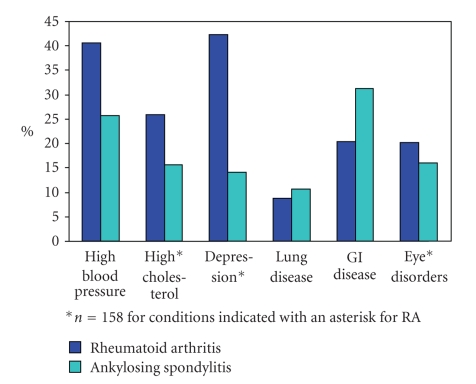
Self-reported comorbidities in ankylosing spondylitis (*n* = 198) and rheumatoid arthritis (*N* = 624*).

To put the high level of baseline comorbidity into context, Figure 2 shows comparable data for ARAD participants with rheumatoid arthritis (RA) commencing biologics. Despite the younger age of the AS cohort (mean age 45.1 (12.3) versus 57.0 (12.5) years for the RA cohort, *P* = .001) the prevalence of at least one comorbid condition was comparable [27] and it is also comparable to the reported baseline comorbidity of RA patients commencing bDMARDs elsewhere [23, 26].

The significantly greater disease activity at commencement of bDMARDs in our AS cohort compared with RCTs [11, 14–16] most likely reflects the stringent PBS requirements for approval of bDMARDs for AS in Australia. Our patients also appear to have greater disease activity at bDMARD commencement than patients commencing bDMARDs in routine care in other settings. For example, a Spanish study of patients with AS commencing biologic therapy reported mean BASDAI scores of 4.5 (versus 7.1 in our study) [45].

Comparable efficacy between RCTs and clinical practice is hardly ever achieved due in part to patient selection, differences in comedications and comorbidities and treatment adherence [46]. Participants in RCTs are likely to be different in some important respects to individual patients seen in clinical practice since minority groups, older individuals, and those at risk of adverse events may be deliberately excluded. The differences we observed may also have been accentuated by the lack of available treatment options prior to the introduction of bDMARD therapy for AS. Over time, it is likely that the average age and disease duration of those prescribed bDMARDs will diminish as newly diagnosed patients who fulfil criteria are treated earlier.

Two of the four trials excluded participants with a history of malignancy [11, 15] and a third excluded patients with an active malignancy in the 5 years prior to the study [16]. The fourth trial did not specify malignancy as an exclusion criterion, and no details regarding presence/absence of malignancy in participants at baseline were reported [14]. We found that 24 (6.8%) AS patients commencing bDMARD therapy in our study had a verified history of malignancy including 15 with nonmelanoma skin cancer and 4 with melanoma. The true prevalence of nonmelanoma skin cancers is likely to be higher but this data is not captured by Australian state registries and we only included self-reported cancers that we could verified by pathology or doctor report.

Available data on risk of malignancy in AS are limited and mainly confined to the increased cancer risk observed in patients with AS subjected to radiation treatment [47]. Recent Swedish population-based studies have not found an increased risk of lymphoma or malignancies overall [48, 49]. It is also unknown whether biologic therapy confers any increased risk of malignancy or recurrence of malignancy in AS. These risks may only be determined by careful long-term comparisons of exposed and unexposed populations.

There are several potential limitations of our study. While we were able to verify the validity of self-reported malignancies, we did not verify the validity of self-report of comorbid conditions. As reported previously, ARAD data are derived predominantly from patient questionnaire [17]. At the time that ARAD was established, there were concerns about the administrative burden of applying for biological therapy and rheumatologists were asked to provide a minimum of information at baseline only. Nevertheless, baseline comorbidities reported by patients with RA in ARAD are comparable to those reported in other studies [18].

A second limitation of our study is that we have not routinely collected data about the presence/absence of extra spinal features of AS such as uveitis, psoriasis, and inflammatory bowel disease, due in part to the fact that ARAD was originally set up for RA. It is likely that some self-reported comorbidities in our study are likely to relate to these extraspinal manifestations of the disease.

## 5. Conclusion

We found that a population-based cohort of patients with AS commencing biological therapy and participating in a national biologics registry already has significant comorbidities. Despite their younger age, the prevalence of comorbidities was comparable to that seen in a population-based RA cohort commencing bDMARDs. These findings have important implications for monitoring patients while on therapy, assessing the long-term health outcomes of bDMARD therapy and attribution of adverse events. We also found that participants in RCTs of bDMARDs for AS were not representative of Australian patients with AS commencing bDMARDs in routine care. As well as being older, having longer disease duration, and more active disease, 6.8% also had a history of verified malignancy. These findings highlight the importance of systematically collecting postmarketing longitudinal outcome data for bDMARDs in routine care.

## Figures and Tables

**Table 1:** Baseline demographic and clinical characteristics of AS patients enrolled in ARAD who have been exposed to biological therapy (*n* = 354)*.

Mean (SD) age, years	45.1 (12.3)
Males (%)	254 (71.8)
Mean (SD) disease duration, years (*n* = 299)	18.5 (12.1)
Mean (SD) delay in diagnosis, years (*n* = 310)	5.5 (7.1)
Concomitant DMARDs, *N* (%)	72 (20.3)
Methotrexate (oral or IM)	50 (14.1)
Salazopyrin	22 (6.2)
Leflunomide	5 (1.4)
Currently taking prednisolone, *N* (%)	42 (11.9)
Smoking history, *N* (%)	
Current	61 (17.2)
Past	98 (27.7)
Never	195 (55.1)
Alcohol consumption, *N* (%)	
Daily	62 (17.5)
Sometimes	217 (61.2)
Never	75 (21.2)
Mean (SD) ESR, mm/hr (*n* = 274)	35.4 (24.8)
Mean (SD) CRP, mg/L (*n* = 300)	31.8 (31.9)
BASDAI (0–10, 0 = best) (*n* = 301)	7.6 (4.5)
Mean (SD) S-HAQ score (0–3, 0 no = disability)**	0.86 (0.60)
Mean (SD) AQoL score (0-1, 1 = full health)**	0.55 (0.25)
SF-36 score**	
Physical component	36.2 (10.6)
Mental component	45.1 (11.1)

*unless otherwise indicated

**n = 198 in those prior to or within 6 months of commencing bDMARDS

**Table 2:** Self-reported comorbid conditions among ankylosing spondylitis patients commencing biological therapy (*n* = 198).

Condition	*N* (%)
Gastrointestinal disease	62 (31.3)
Hypertension	51 (25.8)
Eye disorder	32 (16.1)
Hypercholesterolaemia	31 (15.6)
Depression	28 (14.1)
Anaemia or other blood disorder	28 (14.1)
Lung disease	21 (10.6)
Neurological disorder	18 (9.0)
Osteoporosis	17 (8.6)
Alcohol and drug	13 (6.6)
Other heart disease	13 (6.5)
Liver disease	7 (3.5)
Thyroid disorder	7 (3.5)
Diabetes Mellitus	7 (3.5)
Cerebrovascular accident	5 (2.5)
Kidney disease	4 (2.0)
Angina	4 (2.0)
Mental illness other than depression	4 (2.0)
Tuberculosis	2 (1.0)

**Table 3:** Comparison of ARAD data with data from selected trials of biological disease modifying therapies for AS.

Study (year) Country	Oldroyd et al. * *	van der Heidje et al. (2006) Netherlands [11]		Davis Jr. et al. (2003) USA [14]		Van der Heidje et al. (2005) Netherlands [15]		Breban et al. (2008) France [16]	
Design	Registry	RCT		RCT		RCT		RCT	

Inclusion criteria	In Australia, the PBS criteria for prescription of bDMARDS for AS are: fulfilment of modified NY criteria and failure to respond over 3/12 to at least 2 NSAIDs & an exercise program. (Failure to respond = BASDAI ≥4 and ESR >25 mm/hour and/or CRP >10.0 mg/L)	(1) ≥18 years of age(2) Fulfilment modified New York criteria and active AS defined as 2 of: BASDAI score ≥4 (0–10, 0 = best), morning stiffness ≥1 hour, VAS for total back pain (0–10, 10 = worst) ≥4; inadequate response to NSAIDS; and have failed at least one DMARD		(1) 18–70 year old(2) Fulfilment modified New York criteria and active AS defined as: score of ≥30 mm for morning stiffness ( average of 2 scores on a 100 mm VAS); and scores of ≥30 mm for 2 of the following: patients global assessment (100 mmVAS), back pain, average of 2 VAS scores for night and total back pain and BASFI		(1) Fulfilment modified New York criteria for at least 3 months prior to screening with BASDAI score of ≥4 (0–10, 0 = best) and spinal pain assessment score ≥4 (VAS 0–10 cm); and normal chest radiograph within 3 months prior to randomization and negative test for latent TB or adequate screening for latent TB		(1) ≥18 years(2) fulfilment Modified New York criteria and ≥1 of the following: serum CRP more than twice the upper limit of normal; positive findings on MRI of the spine or sacroiliac joints or a vascularised enthesitis on power Doppler ultrasound; BASDAI ≥3 (0–10, 0 = best) and axial pain (2nd item on BASDAI) ≥3	

Exclusion criteria	Nil	Previous receipt of anti-TNF therapy, cyclosporine, azathioprine, or DMARDS (other than sulfasalazine (≤3 gm/day), methotrexate (≤25 mg/wk), hydroxychloroquine (≤400 mg/day), prednisone (≤10 mg/day) and NSAIDS) at any time; receipt intraarticular corticosteroid injections within 4 weeks of baseline; clinically active TB; history of recent infections requiring antibiotic treatment; hepatitis; HIV; significant history of cardiac, renal, neurological, psychiatric, endocrine, metabolic, or hepatic disease; history of demyelinating disease or multiple sclerosis; history of cancer or lymphoproliferative disease (except BCC or SCC or localised cancer of the cervix in situ)		Complete ankylosis (fusion) of the spine on radiographic assessment; previous TNF inhibitor therapy; serious infection (associated with hospitalization or intravenous antibiotics) within 4 weeks before screening; pregnancy; any DMARDS within 4 weeks of baseline evaluations (other than hydroxychloroquine, sulfasalazine, methotrexate in stable doses); unstable dosages of NSAIDS or prednisone (≤10 mg/day)		Total ankylosis spine (defined by syndesmophytes present on the lateral views of spinal radiographs at all intervertebral levels from T6 through S1); any other inflammatory rheumatic disease; fibromyalgia; a serious infection within 2 months prior to randomization; TB; (active or latent) or recent contact with a person with active TB; an opportunistic infection within 6 months of screening; hepatitis; HIV; a transplanted organ; malignancy; multiple sclerosis; congestive heart failure; taking sulfasalazine or methotrexate within 2 weeks prior to screening; systemic corticosteroids within 1 month prior to screening; anti-TNF therapy other than infliximab within 3 months prior to screening; infliximab at any other time prior to screening; taking DMARDS other than sulfasalazine or methotrexate within 6 months prior to screening or cytotoxic drugs within 12 months of screening		Pregnancy or breastfeeding; vaccination with live organism during month prior to study entry; an infection at study entry or had any episode of serious infection within 3 months prior to study entry; active malignancy in 5 years prior to study entry; addicted to drugs or alcohol; severe chronic concomitant disease; receipt of investigational drug in 3 months prior to study entry, or had received any known TNF inhibitor therapy in the past; failure to discontinue DMARDS such as sulfasalazine, MTX, hydroxychloroquine, intramuscular gold, thiol compound, cyclosporine and intravenous biphosphonate ≥4 weeks prior to enrolment; unstable dose of NSAIDS and corticosteroids for ≥4 weeks before enrolment.	
Intervention	None	Adalimumab 40 mg 1x per 2 weeks for 24 weeks	Control	Etanercept 25 mg 2x per week for 24 weeks	Control	Infliximab 5 mg/kg at week 0, 2, 6, 12, 18, over 24 weeks	Control	Infliximab “continuous” 5 mg/kg every 6 weeks for 54 weeks	Infliximab “on demand” 5 mg/kg upon relapse +/− MTX for 54 weeks
Patient numbers	354	208	107	138	139	201	78	124	123
Age, years, mean (SD)	45.1 (12.3)*	41.7 (11.7)	43.4 (11.3)	42.1 (24–70)*α*	41.9 (18–65)*α*	40 (32.0–47.0)^^^	41 (34.0–47.0)^^^	41.4 (12.3)	41.3 (10.3)
Males, *N* (%)	254 (71.8)	157 (75.4)	79 (73.8)	105 (76.1)	105 (75.5)	157 (78.1)	68 (87.2)	93 (60.5)	95 (77.2)
Disease duration, years, mean (SD)	18.5 (12.1)*	11.3 (9.9)	10.0 (8.3)	10.1 (0–30.7)*α*	10.5 (0–35.3)*α*	7.7 (3.3–14.9)^^^	13.2 (3.7–17.9)^^^	14.6 (10.5)	15.1 (9.3)
HLA-B27 positive *N* (%)	*N*/A	163 (78)	85 (79)	108 (84)	109 (84)	173 (87)	69 (89)	80 (65)	81 (66)
Swollen joint count (0-44 joints) mean (SD)	*N*/A	1.5 (3.3)	1.4 (2.8)	*N*/R	*N*/R	0 (0-1)	0 (0-1)	*N*/A	*N*/A
History of uveitis, *N* (%)	*N*/A	68 (32.7)	27 (25.2)	39 (28)	43 (31)	72 (35.8)	25 (32.1)	33(27)	43 (35)
History of psoriasis, *N* (%)	*N*/A	68 (32.7)	27 (25.2)	11 (8)	1 5(11)	16 (8.0)	5 (6.4)	20 (16)	13 (11)
History of IBD, *N* (%)	*N*/A	16 (7.7)	17 (15.9)	7 (5)	6(4)	13 (6.5)	6 (7.7)	12 (10)	12 (10)
BASDAI (0–10, 0 = best), mean (SD)	7.6 (4.5)*	6.3 (1.7)	6.3 (1.7)	58.1 (1.5)*β* ^#^	59.6 (SEM 1.4)*β* ^#^	6.6 (5.3–7.6)^^^	6.5 (5.2–7.1)^^^	6.2 (1.5)	6.2 (1.3)
Patient's assessment of pain in past week (0-10, 0 = none), mean (SD)	44.8 (28.1) (out of 100)	6.4 (2.1)	6.7 (2.2)	*N*/R	*N*/R	*N*/R	*N*/R	6.9 (1.9)	6.7 (1.8)
Patient's global assessment disease activity in the past week (0–10, 0 = none), mean (SD)	43.2 (28.0)(out of 100)	6.3 (2.2)	6.5 (2.0)	62.9 (out of 100)	62.9 (out of 100)	6.9 (5.7–8.0)^^^	6.7 (5.8–7.7)^^^	7.4 (2.9)	7.5 (1.5)
CRP (mg/dl) mean (SD)	31.8 (31.9) mg/L*	1.8 (2.2)	2.2 (2.9)	1.9 (0.2)*β*	2.0 (0.2)*β*	1.5(0.7–3.2)^^^	1.7 (0.7–3.3)^^^	33 (27) mg/L	29 (21) mg/L
ESR (mm/hour) mean (SD)	35.4 (24.8)*	*N*/A	*N*/A	25.9 (1.8)*β*	25.4 (1.9)*β*	*N*/A	*N*/A	37 (25)	32 (21)
SF-36 Physical Component score Mental Health Component score	35.4 (10.3) 45.1 (11.2)	*N*/R	*N*/R	*N*/R	*N*/A	28.8 (23.8–33.7)^^^47.6 (37.6–54.9)^^^	30.1 (24.9–36.2)^^^45.0 (33.7–55.5)^^^	33 (7) 34 (10)	31 (7) 36 (10)
AQoL (0-1, 1 = full health) mean (SD)	0.55 (0.25)	*N*/R	*N*/R	*N*/A	*N*/A	*N*/A	*N*/A	*N*/A	*N*/A
S-HAQ (0–3, 0 = no disability), mean (SD)	0.86 (0.60)	*N*/R	*N*/R	*N*/A	*N*/A	*N*/A	*N*/A	*N*/A	*N*/A
Concomitant DMARDS *n* (%)	72 (20.3)	40 (19.2)	22 (20.6)	44 (32)	43 (31)	0	0	0	0
Methotrexate, *N* (%)	50 (14.1)	26 (12.5)	15 (14.0)	15 (11)	17 (12)	0	0	0	0
Sulfasalazine, *N* (%)	22 (6.2)	20 (9.6)	8 (7.5)	29 (21)	30 (2)	0	0	0	0
Leflunomide, *N* (%)	5 (1.4)	0	1 (0.9)	*N*/R	*N*/R	0	0	0	0
Prednisolone, *N* (%)	42 (11.9)	25 (12.0)	6 (5.6)	20 (14)	18 (13)	0	0	*N*/R	*N*/R
Comorbidities									
Gastrointes-tinal disease, *N* (%)	62 (31.3)	*N*/A	*N*/A	*N*/A	*N*/A	*N*/A	*N*/A	*N*/A	*N*/A
Hypertension, *N* (%)	51 (25.8)	*N*/A	*N*/A	*N*/A	*N*/A	*N*/A	*N*/A	*N*/A	*N*/A
Eye disorders, *N* (%)	32 (14.1)	*N*/A	*N*/A	*N*/A	*N*/A	*N*/A	*N*/A	*N*/A	*N*/A

*P < .001 for difference between mean (SD) in ARAD versus weighted mean (SD) in trials

*α* means (range)

*β* means (SEM)

^#^BASDAI score reported as mean (standard error of the mean) and range 0–100 for this; study ^^^median (IQR)

*N*/A = not measured

*N*/R= measured but not reported.
